# Modulating ion channels with nanobodies

**DOI:** 10.1016/j.synbio.2025.02.005

**Published:** 2025-02-18

**Authors:** Sher Ali, Ashley Suris, Yun Huang, Yubin Zhou

**Affiliations:** a Center for Translational Cancer Research, Institute of Biosciences and Technology, Texas A&M University, Houston, TX 77030, USA; b Center for Epigenetics and Disease Prevention, Institute of Biosciences and Technology, Texas A&M University, Houston, TX 77030, USA; c Department of Translational Medical Sciences, College of Medicine, Texas A&M University, Houston, TX 77030, USA

**Keywords:** Nanobody, Antibody engineering, Ion channels, Immunotherapy, Synthetic biology, Therapeutics

## Abstract

Ion channels play instrumental roles in regulating membrane potential and cross-membrane signal transduction, thus making them attractive targets for understanding various physiological processes and associated diseases. Gaining a deeper understanding of their structural and functional properties has significant implications for developing therapeutic interventions. In recent years, nanobodies, single-domain antibody fragments derived from camelids, have emerged as powerful tools in ion channel and synthetic biology research. Their small size, high specificity, and ability to recognize difficult-to-reach epitopes offer advantages over conventional antibodies and biologics. Furthermore, their resemblance to the variable region of human IgG family III reduces immunogenicity concerns. Nanobodies have introduced new opportunities for exploring ion channel structure-function relationships and offer a promising alternative to conventional drugs, which often face challenges such as off-target effects and toxicity. This review highlights recent progress in applying nanobodies to interrogate and modulate ion channel activity, with an emphasis on their potential to overcome current technical and therapeutic limitations.

## Graphical abstract

**Figure:**
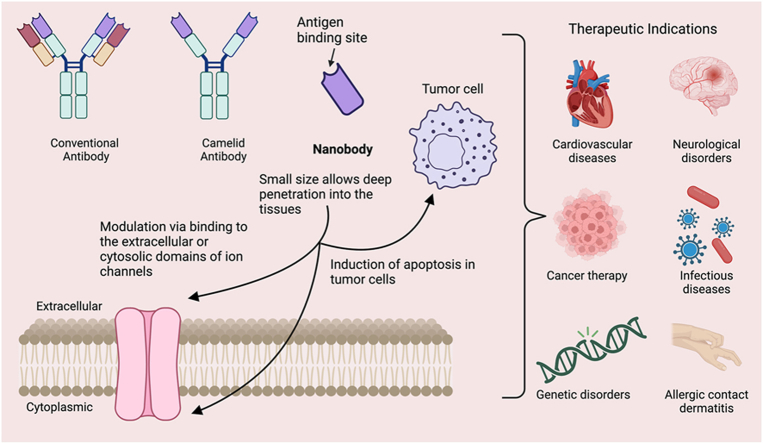

## 1 Introduction

Voltage- and ligand-gated ion channels constitute a diverse family of transmembrane proteins that control ion flow across membranes through gated pores. These channels are essential for regulating a wide range of biological processes, including hormone secretion, cell proliferation, cell migration, programmed cell death, neuronal excitability, muscle contraction, signal transduction, blood pressure regulation, and gene transcription [1,2]. The human genome contains hundreds of genes that encode ion channels, which can be broadly categorized as either voltage-gated or ligand-gated, based on the mechanisms regulating pore opening and closing [3]. Genetic defects in genes encoding ion channels are linked to a large number of channelopathies that affect the cardiac, neurological and immune systems [[4], [5], [6]]. For example, neuromuscular disorders such as Lambert-Eaton myasthenic syndrome and Isaacs' syndrome arise from autoantibodies that target voltage-gated calcium (Ca^2+^) and potassium (K^+^) channels, respectively [[7], [8], [9]]. Similarly, mutations in the gene encoding the Ca^2+^ release-activated Ca^2+^ (CRAC) channel, *ORAI1*, and its activator stromal interaction molecule 1 (*STIM1*), are implicated in severe combined immunodeficiency (SCID), tubular aggregate myopathy (TAM), and Stormorken syndrome [10,11]. Additionally, impaired degradation of renal epithelial Na^+^ channels leads to Liddle syndrome [12], which is characterized by hypertension with hypokalemic metabolic alkalosis, suppressed aldosterone secretion, and hyporeninemia. The pivotal role of ion channels in regulating a multitude of cellular functions and their involvement in a broad spectrum of diseases underscores their significance as valuable therapeutic targets.

Over the past five decades, remarkable progress has been made in the pursuit of small molecules targeting ion channels, which substantially advances the treatment of neurological and cardiovascular disorders, as well as pain management [1]. These efforts, driven by high-throughput compound screening and serendipitous discoveries, have given rise to novel classes of ion channel-targeting therapeutics [1,13]. For instance, small molecule blockers of high-voltage-activated Ca^2+^ channel (HVACC), such as benzothiazepines, phenylalkylamines and dihydropyridines, have become indispensable therapeutics in the clinical management of conditions like cerebral vasospasm, hypertension, cardiac arrhythmias, Parkinson's disease, and epilepsy [14,15]. More recently, emerging evidence suggests that ion channel blockers may also play a role in combating multidrug resistance in cancer, thus expanding their therapeutic potential [16,17].

Despite these advances, small-molecule modulators of ion channels still face several challenges. These include off-target effects, difficulty in achieving selectivity across ion channel subtypes or isoforms, and plasma half-lives limited to the scale of minutes to hours, which constrain their sustained efficacy and specificity. Furthermore, prolonged use of small-molecule inhibitors has been linked to the development of therapeutic resistance [18]. To address these challenges, monoclonal antibodies (mAbs) have emerged as a transformative class of therapeutics, particularly in oncology and autoimmune diseases [19]. However, mAbs present their own limitations, primarily due to their large size (∼150 kDa), which leads to reduced tissue penetration and suboptimal biodistribution [20]. Nanobodies (Nbs), derived from camelid antibodies, have garnered increasing attention as highly modular and versatile alternative ion channel modulators [21]. Owing to their small size and extraordinary binding specificity, Nbs offer several distinct advantages over mAbs and small molecules. Their compact structure allows for superior tissue penetration and the ability to access epitopes that are often inaccessible to larger mAbs. Additionally, their high specificity and tunable affinities make them particularly well-suited for targeting ion channels, thereby providing solutions to the common challenges of off-target effects and therapeutic resistance observed with small molecules.

Nanobodies are not only more modularly adaptable but can also be flexibly engineered with greater precision and tunability to modulate specific ion channel subtypes, hence addressing the issue of selectivity that has historically hindered other therapeutic approaches. These properties make nanobodies an ideal platform for developing next-generation therapies for ion channel-related diseases. This review will focus on recent advances in the use of nanobodies to modulate ion channel activity, exploring their physiological and pathophysiological roles, and highlighting their potential as next-generation therapeutics for ion channel-related diseases.

## 2 Nanobodies as promising tools for research and theragnostic applications

**Fig. 1:**
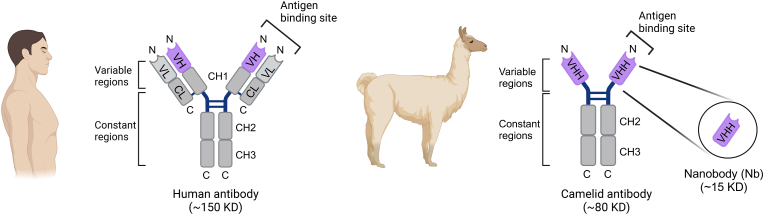
Schematic illustration of human and camelid antibodies and their fragments. In a typical mammalian antibody, the light chain consists of one variable (VL, light grey) and one constant (CL, dark grey) domain, while the heavy chain contains one variable (VH, purple) and three constant (CH1 to CH3, dark grey) domains. The antigen binding fragment (F_ab_) is formed by the paring of VL and VH domains. In contrast, the camelid antibody exists as a homodimer with heavy chains only. The isolated variable region of the camelid antibody provides a functional single-domain antibody (VHH), commonly known as a nanobody.

Nanobodies are a class of compact single-domain antibodies derived from a unique heavy-chain-only antibody (HCAb) naturally produced by camelid species [22]. In contrast to conventional antibodies, which contain two identical heavy-chain (CH) and light-chain (CL) polypeptides that pair to form a stably folded protein, HCAbs lack both the CL and first constant domain (CH1) (Fig. 1) [23,24]. A nanobody consists of the isolated HCAb variable domain, designated VHH, which binds to its cognate antigen and functions as the equivalent to the antigen-binding fragment (F_ab_) of conventional antibodies. With a molecular weight of approximately 15 kDa, nanobodies are only one-tenth the size of conventional antibodies. Similar to the variable regions of traditional antibodies, nanobodies contain three hypervariable complementarity-determining regions (CDRs) linked together by highly conserved beta-sheet framework regions [25]. The elongated third CDR, paired with its compact size, allows for the adoption of unique conformations and the ability to reach previously inaccessible target epitopes [[26], [27], [28]]. Notably, the framework regions of nanobodies exhibit high homology to human family III heavy chain domains, thereby reducing the potential risk of immunogenicity when administered to humans [25]. Additionally, nanobodies have demonstrated enhanced solubility and stability as compared to traditional antibodies and antibody fragments [29]. These favorable attributes fuel interest in the utilization of nanobodies for research, diagnostic, and therapeutic applications, including ion channel modulation (Table 1).

**Table 1:** Key information on published nanobodies targeting ion channels described in this review.

Nb	Target	Modality	Method	Immunogen	Mode of action	Therapeutic indications	K_D_ (nM)	Ref
13A7-HLE	mP2X7	Antagonist	Llama immunization/phage display	mP2X7 transfected HEK-293 cells or with P2X7 cDNA expression vector	Blockade of ATP-induced Ca^2+^ influx via P2X7	Inflammatory diseases: glomerulonephritis and allergic contact dermatitis	ND	[33]
14D5-HLE	mP2X7	Agonist	Llama immunization/phage display	mP2X7 transfected HEK-293 cells or with mP2X7 cDNA expression vector	Reducing ATP threshold required for the activation of P2X7	Pathological conditions such as cancer or infections with intracellular parasites	ND	[33]
Dano1	hP2X7	Antagonist	Llama immunization/phage display	hP2X7 transfected HEK-293 cells or with hP2X7 cDNA expression vector	Blockade of ATP-induced IL-1β release	Glomerulonephritis, lupus nephritis, and acute dermatitis	ND	[33]
Nb.F3 (Ca_V_-aβlator)	Ca_V_1.2 β subunit	Antagonist	Llama immunization/phage display	Purified β_1_ and β_3_ proteins from HEK-293 cells overexpressing Ca_V_β_1b_ and Ca_V_β_3_	Cytosolic β-domains	Pain, hypertension, cardiac arrhythmias, epilepsy, and Parkinson's disease	13.2 ± 7.2	[42]
Nb.E8 (Chisel-1)	Ca_V_1.2 β1 subunit	Antagonist	Llama immunization/phage display	Purified β_1_ and β_3_ proteins from HEK-293 cells over expressing Ca_V_β_1b_ and Ca_V_β_3_	Cytosolic β_1_-domain	Cardiovascular and neurological diseases	ND	[44]
Nb.C1	hASIC1a	Antagonist	Llama immunization /phage display	hASIC1a transfected HEK-293T cells	Extracellular domain of the channel	Potential to be used in neurological disorders	ND	[52]
Nb17, Nb82	Na_V_ 1.4 or Na_V_ 1.5	Antagonist	Llama immunization/phage display	Purified Na_V_1.4 protein fragment in complex with Calmodulin (CaM)	Cytosolic domain (CT)	Human genetic diseases caused by mutation in the CT, e.g., generalized epilepsy with febrile seizures, hypokalemic periodic paralysis, myotonia, long-QT syndrome, and Brugada syndrome	Nb17: 41.1 ± 9. 9; 60.5 ± 5.80 Nb82: 50.2 ± 8.87; 63.2 ± 6.75	[58]
VHH-D9-scTRAIL	mK_V_ 10.1	Antagonist	Llama immunization/phage display	mK_V_10.1 derived antigen	Extracellular/Induce apoptosis into the tumor cells	Human pancreatic cancer	78	[62]
A0194009G09	K_V_1.3	Antagonist	Immunoglobulin based protein engineering	Immunoglobulin recognizing the first extracellular loop of K_V_1.3	Extracellular/C-type inactivation of channel	Autoimmune diseases, such as multiple sclerosis, type-1 diabetes mellitus, rheumatoid arthritis and psoriasis.	ND	[63]

ND, not determined; P2X7, purinergic P2X receptor 7; Ca_V_, voltage-gated Ca^2+^ channel; K_V_, voltage-gated K^+^ channel; ASIC, acid-sensing ion channel; IL, interleukin.

## 3 Nanobodies targeting ion channels

### 3.1 P2X7 ion channels

P2X7 is a non-selective adenosine 5-triphosphate (ATP)-gated cation channel expressed in macrophages and regulatory T-cells that permits calcium (Ca^2+^) and sodium (Na^+^) influx and efflux of potassium ions (K^+^). ATP released from cells under stress during inflammation and tumor development triggers the activation of P2X7 channels. This activation initiates a proinflammatory signaling cascade and leads to the release of cytokines, including interleukin-1β (IL-1β) [30]. As such, these channels are attractive therapeutic targets for a wide range of inflammatory diseases, including multiple sclerosis, glomerulonephritis, and chronic pain [31,32].

**Fig. 2:**
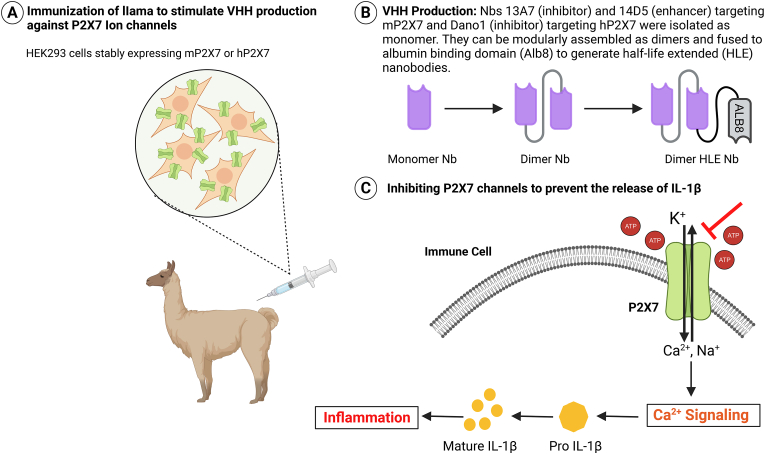
Schematic diagram showing the generation of P2X7-targeting nanobodies. **A.** Llama was immunized with human and mouse P2X7-transfected HEK293 cells to generate anti-P2X7 nanobodies. **B.** Nanobodies were isolated as monomers and re-engineered into dimer along with Alb8. **C.** Nbs inhibit ATP-activated P2X7 channels, which are involved in inflammatory response by inducing Ca^2+^ influx and ultimately causing the production of pro-inflammatory cytokines, such as IL-1β.

Danquah et al. developed potent nanobodies against mouse and human P2X7 ion channels from immunized llamas (Fig. 2A). Nanobody 13A7, the most potent murine P2X7 blocker (IC_50_: 12 nM), could effectively inhibit ATP-induced whole-cell currents and Ca^2+^ influx. In contrast, nanobody 14D5 (IC_50_: 6 nM), strongly enhanced ATP-induced whole cell currents and Ca^2+^ influx in mouse P2X7-expressing cells [33]. The binding affinities and potencies were improved by the generation of a dimeric NB, involving the fusion of two single domains via a flexible peptide linker. Further, generation of a dimeric half-life extension (HLE) version through the fusion of homodimers of 13A7 or 14D5 to an anti-albumin antibody moiety (Alb8), extended both the half-life from hours to days, and the duration of P2X7 modulation (Fig. 2B). Systemic administration of dimer-HLE nanobody 13A7 ameliorated inflammation in glomerulonephritis and allergic contact dermatitis mouse models [33].

Similarly, the nanobody Dano1, which targets human P2X7 (hP2X7), effectively blocked ATP-induced Ca^2+^ influx in hP2X7-expressing cells and inhibited the release of proinflammatory cytokines with up to 1000-fold greater potency than small-molecule inhibitors. Furthermore, Dano1 demonstrated high specificity with no crosstalk between species and orthologs, positioning it as a strong candidate for translational development. Given the central role of IL-1β inhibition in managing chronic inflammatory diseases, targeting P2X7 with nanobodies presents an attractive alternative approach to suppress IL-1β (Fig. 2C). Another study used adeno-associated virus (AAV) delivery to produce an endogenous heavy-chain antibody consisting of a non-modulatory anti-P2X7 nanobody fused to murine IgG2a for *in vivo* cell depletion [34]. This study establishes a proof-of-concept for utilizing nanobodies as effective tools for ion channel research.

Of particular interest, anti-P2X7 nanobodies were able to cross the blood-brain barrier (BBB) and block P2X7 channels in microglia in mouse models of systemic inflammation when administered intravenously at high doses or intracerebrally at low doses [35]. Endogenous production of nanobodies following AAV administration enabled sustained channel blockade over the longer term. Furthermore, anti-P2X7 nanobody treatment reduced the risk of ischemic tissue damage in mice following induced stroke [36]. Collectively, these findings highlight the ability of nanobodies to cross the BBB and support their potential as therapeutics for brain inflammation.

### 3.2 Voltage-gated Ca^2+^ channels (VGCCs)

VGCC play a crucial role in excitable cells, mediating processes such as muscle contraction, hormonal secretion, and neurotransmitter release [15,37]. As a result, they serve as promising therapeutic targets for a myriad of cardiovascular and neurological diseases. VGCCs exist as multi-subunit complexes, with seven distinct subtypes (Ca_V_1.1–Ca_V_1.4 and Ca_V_2.1–Ca_V_2.3) comprising an α_1_ voltage-sensor, selectivity filter, and pore-forming subunit assembled with auxiliary proteins which include β, α_2_-δ, and γ [14,38]. There are four Ca_V_β subunit isoforms (Ca_V_β_1_–Ca_V_β_4_) encoded by distinct genes [39]. Auxiliary proteins involved in the trafficking, gating, and modulation of VGCCs are increasingly recognized as potential therapeutic targets for treating VGCC-related diseases. For example, gabapentin targets α_2_-δ subunits for the treatment of neuropathic pain and epilepsy [40,41]. Previous studies have shown that the association of pore-forming subunit α_1_ with β is essential for the formation of functional VGCC. Disrupting the α1-β interaction has been pursued as a strategy to inhibit VGCC activity [14].

**Fig. 3:**
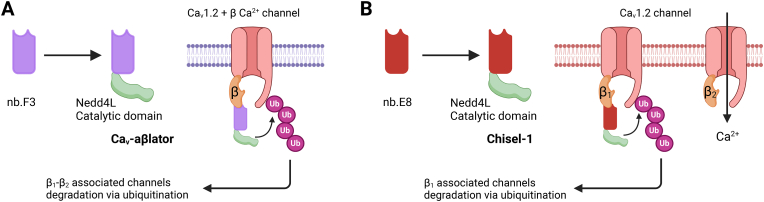
Nanobody-mediated degradation of Ca_V_ channels. **A.** The anti-Ca_V_β nanobody nb.F3 is fused with the catalytic HECT domain of the Nedd4L E3 ubiquitin ligase to generate Ca_V_-aβlator. Ca_V_-aβlator is capable of catalyzing the ubiquitination of the Ca_V_1.2/Ca_V_β Ca^2+^ channel, ultimately leading to their functional inhibition. **B.** The anti-Ca_V_β1 nanobody nb.E8 is fused with the catalytic HECT domain of Nedd4L to yield Chisel-1. Chisel-1 catalyzes the ubiquitination of the Ca_V_1.2/Ca_V_β1 Ca^2+^ channel to suppress the channel activity via proteasomal degradation.

Morgenstern et al. developed a llama-derived anti-Ca_V_β nanobody (nb.F3) fused to an E3 ubiquitin ligase to inhibit Ca_V_1.2 channel activity [42]. Nb.F3 was shown to bind to β1 through β4 subunits in transfected cells and assemble into Ca_V_ channel complexes without disrupting channel function. To achieve inhibition, Nb.F3 was fused with the catalytic Homologous to the E6-APC Carboxyl Terminus (HECT) domain of an E3 ubiquitin ligase, neural precursor cell expressed developmentally downregulated gene 4-like (Nedd4L) (Fig. 3A). The resultant Ca_V_-aβlator construct blocked whole-cell currents in diverse reconstituted Ca_V_1.2 channels in HEK293 cells, as well as native mammalian cardiomyocytes, dorsal root ganglion neurons, and pancreatic β cells [42]. The Ca_V_-aβlator approach introduced a versatile strategy for modulating protein complexes and demonstrated therapeutic potential. Subcutaneous AAV delivery of Ca_V_-aβlator reduced hyperalgesia and Ca^2+^ spike recordings in mice subjected to spare nerve injury, highlighting its neuropathic therapeutic efficacy. These studies showcase the potential of the Ca_V_-aβlator as a platform for post-translational inactivation of ion channels through the ubiquitin-proteasome system [43].

Although the Ca_V_-aβlator demonstrates therapeutic potential, it lacks specificity for individual Ca_V_β subtypes. Ca_V_β1–CaVβ4 share overlapping functions, such as shifting the voltage dependence of channel activation in a hyperpolarizing direction, facilitating the co-expression of the α1 subunit, and increasing channel open probability (*P*_*o*_). However, each β subunit subtype imparts unique properties to VGCCs, including distinct rates of inactivation and steady-state inactivation, thereby offering an opportunity to improve the specificity of Ca_V_-aβlator. The development of Chisel-1 demonstrated potent and specific inhibition Ca_V_β_1_ subunit through targeting the unique SH3 domain and promoting targeted ubiquitination, thus reducing the *P*_*o*_ of the channel and reducing channel surface density (*N*) (Fig. 3B) [44]. Chisel-1 represents the first platform capable of selectively ablating VGCC function based on the isoform identity of the auxiliary subunit. Given that many other ion channels, beyond VGCCs, also consist of pore-forming proteins paired with auxiliary subunits of various isoforms, Chisel-1 demonstrates the potential of nanobody-based tools to selectively probe and modulate the functions of distinct ion channel subunit isoforms.

The introduction of a genetically encoded enhancer of Ca^2+^ channels—L-type (GeeC_L_) facilitated the enhancement of L-type Ca^2+^ channel activity in a targeted manner by leveraging a fusion of a high-affinity nanobody directed at Ca_V_ complex subunits with the minimum effector domain from the Ca_V_1.2 modulating leucine-rich repeat-containing protein 10 (Lrrc10) [45]. GeeC_L_ selectively increased channel open probability and enabled precise modulation of neuronal excitation-transcription coupling and cardiomyocyte Ca^2+^ influx. Applications of this technology could include restoring impaired Ca^2+^ signaling in Rett syndrome neurons, demonstrating its therapeutic potential and opening new avenues for isoform-specific VGCC research [46].

### 3.3 Acid-sensing ion channels (ASICs)

ASICs are sodium channels activated by protons. These channels are expressed in neurons of both central and peripheral nervous systems and play roles in ischemia-induced neuronal injury, as well as the modulation of pain, fear, and addiction [46]. Four ASIC genes (*ASIC1*–*4*) encode six distinct subunits: ASIC1a, ASIC1b, ASIC2a, ASIC2b, ASIC3, and ASIC4 [47]. These subunits assemble into homo- or heterotrimers to form functional channels, with ASIC1a being the most abundant. ASIC2a deletion in mice eliminates the majority of ASIC-mediated currents [48]. While protons are the canonical ligands for ASICs, their large extracellular loops permit the binding of non-proton ligands, such as venoms from the Texas coral snake (Micrurus tener tener, MitTx) and the tarantula *Psalmopoeus cambridgei* (PcTx1) [[49], [50], [51]]. MitTx, a heterodimer composed of a 60-residue α-subunit and a 119-residue β-subunit, acts as a selective agonist for ASICs, whereas PcTx1, a 40-residue peptide, potently and selectively inhibits hASIC1a.

**Fig. 4:**
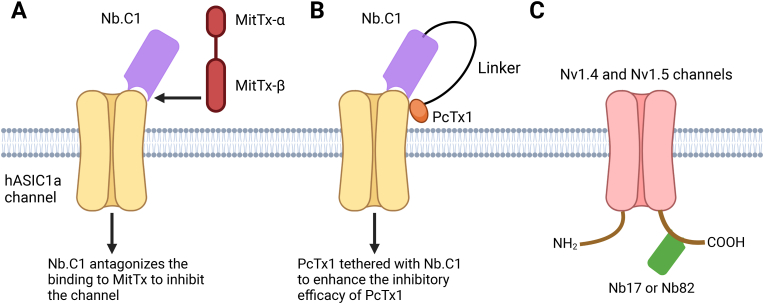
Nanobody-mediated direct modulation of ASICs or Na_V_ channels. **A.** Cartoon illustrating how Nb.C1 interferes with the binding of a venom toxin from the Texas coral snake (MitTx), thereby inhibiting MitTx -activated hASIC1a channels. **B.** A Nb.C1-PcTx1 fusion protein is engineered to provide more precise hASIC1a targeting with an enhanced analgesic effect. **C.** Nb17 and Nb82 specifically target the C-terminal regions of the 1.4 and 1.5 isoforms of Na_V_ channels for functional tuning.

Wu et al. developed nanobody Nb.C1, which demonstrated high specificity and affinity for hASIC1a [52]. In HEK293 cells over-expressing hASIC1a, Nb.C1 prevented hASIC1a aggregation and stabilized channel expression, opening avenues for studying channel structure and function. This stabilization also improved hASIC1 sample preparation, enabling cryo-EM analysis and yielding a 2.9 Å resolution structure of the hASIC1-Nb.C1 complex in the closed conformation. This represents a proof-of-concept for using nanobody stabilization to facilitate structural analysis of ion channels [52]. Interestingly, the Nb.C1 binding site overlaps with that of MitTx, a toxin that induces severe pain by activating ASIC1a channels, but not with the ASIC1a-inhibitory PcTx1 toxin (Fig. 4A). This suggests that Nb.C1 could serve as a low-cost time-efficient alternative antidote for the pain-producing effects of snakebite venom, potentially eliminating the need for traditional antivenom derived from horses or sheep. Moreover, the distinct binding modes of PcTx1 and Nb.C1 raise the possibility that Nb.C1 can be used as a carrier to deliver PcTx1 specifically to its binding site, thereby minimizing off-target effects and enhancing PcTx1 potency [52]. This strategy could advance the therapeutic potential of PcTx1 as an analgesic or neuroprotectant (Fig. 4B). Congruently, these studies demonstrate the ability of nanobodies to be used to advance structural and functional studies of ion channels and as vehicles for enhancing drug delivery specificity.

### 3.4 Voltage-gated sodium channels

Voltage-gated sodium channels (Na_V_) rapidly respond to changes in membrane potential by allowing Na^+^ influx. These channels play an important role in the generation and propagation of action potentials in excitable tissues such as the heart, muscles, and nerves. Na_V_ cahnnels are heteromultimeric proteins consisting of a single pore-forming α-subunit complexed with one or two small accessory β subunits. The human genome encodes nine distinct genes (*SCN1A*, *SCN2A*, *SCN3A*, *SCN4A*, *SCN5A*, *SCN8A*, *SCN9A*, *SCN10A*, and *SCN11A*) and four β subunit genes (*SCN1B*, *SCN2B*, *SCN3B* *and* *SCN4B*) [53]. While the central and peripheral nervous systems express most isoforms, skeletal and cardiac muscles exhibit a more restricted Na_V_ repertoire [54]. Mutations in Na_V_-encoding genes are associated with a variety of genetic disorders affecting skeletal muscle contraction and the nervous system. These include epilepsy with febrile seizures, myotonia, long-QT syndrome, hypokalemic periodic paralysis, and Brugada syndrome [[55], [56], [57]].

The C-terminal domains of two Na_V_ isoforms, Na_V_1.4 and Na_V_1.5, were selected as antigens for Nb production due to their regulatory roles as the binding sites for channel-interacting proteins [58]. Nb17 and Nb82 were developed to specifically recognize Na_V_1.4 and Na_V_1.5 with nM affinities, but not other isoforms. These nanobodies successfully detected Na_V_ channel expression in mammalian cells and tissues, showcasing their potential utility in the molecular detection, visualization, and capture of Na_V_ channels in cellular and tissue environments.

### 3.5 Voltage-gated potassium channels

Voltage-gated potassium channels (K_V_) are critical for modulating resting and action potentials as well as stabilizing membrane potentials in a variety cell types. K_V_ channels have been implicated in cancer, autoimmune disorders, and inflammatory diseases [59]. K_V_1.3-specific nanobodies characterized by Ablynx exhibited valency-dependent modulatory effects [60]. Monovalent nanobodies demonstrated rapid kinetic profiles, while bivalent nanobodies not only increased avidity but also extended target residence time and blockade duration. Unsurprisingly, the composition of the bivalent nanobody resulted in varied outcomes. Homo-bivalent nanobodies amplified the effects of the monovalent component, whereas hetero-bivalent nanobodies produced mixed phenotypes. A trivalent nanobody further amplified the effects of the monomeric molecule. Among these, a homo-bivalent-HLE version demonstrated the most promise and reduced ear thickness in a delay-type hypersensitivity rat model. These findings underscore the potential of valency-optimized nanobodies for therapeutic development.

K_V_10.1 is frequently overexpressed in cancer cell lines [61]. An anti-K_V_10.1 nanobody, D9, was developed and demonstrated nanomolar affinity [62]. When fused to a single-chain tumor necrosis factor-related apoptosis-inducing ligand (VHH-D9-scTRAIL), the resulting construct induced potent tumor cell apoptosis across multiple cell lines. Remarkably, the VHH-D9-scTRAIL fusion triggered apoptosis more rapidly and effectively than either component alone, underscoring its potential as a novel strategy in cancer therapeutics.

An anti-K_V_1.3 nanobody, A0194009G09, was instrumental in elucidating the conformational changes required for the transition of K_V_1.3 channels from an open-conducting state to an inactivated conformation [63]. By stabilizing the inactivated state of both wild-type K_V_1.3 and two mutants, this nanobody provided valuable insights into the mechanisms of slow channel inactivation. These findings greatly enhance our understanding of K_V_1.3 channel dynamics and advance the development of targeted K_V_1.3 therapeutics.

## 4 Concluding remarks

Nanobodies have emerged as transformative tools for studying and modulating ion channels, providing significant advantages over traditional small molecules and monoclonal antibodies due to their small size, high specificity, and ability to target previously inaccessible epitopes. These unique properties make them highly valuable for studying the structure and function of ion channels. Further optogenetic or chemogenetic engineering of ion channel-targeting nanobody platforms, such as Ca_V_-aβlator and Chisel-1, could offer unprecedented control over processes such as ion channel degradation, subcellular relocalization, and even post-translational modifications [[64], [65], [66], [67]]. Nanobody-based magnetic modulation of the transient receptor potential vanilloid (TRPV) ion channel is currently under investigation [68]. Continued advancements in nanobody development and screening methods are expected to expand the therapeutic and modulatory repertoire for ion channels.

The potential of nanobodies extends beyond basic research by offering innovative therapeutic opportunities for a wide range of ion channel-related diseases, including neurological, cardiovascular, and immunoinflammatory conditions. Recent advancements in nanobody engineering, such as enhanced stability, improved tissue penetration, and tunable affinity, underscore their promise as next-generation therapeutics. By addressing long-standing challenges in ion channel drug development—such as selectivity, off-target effects, and therapeutic resistance—nanobodies represent a novel and versatile platform for both research and clinical applications, with the potential to revolutionize treatments for channelopathies and other diseases involving ion channel dysfunction.

## CRediT authorship contribution statement

**Sher Ali:** Writing – review & editing, Writing – original draft. **Ashley Suris:** Writing – review & editing, Writing – original draft. **Yun Huang:** Writing – review & editing, Funding acquisition, Conceptualization. **Yubin Zhou:** Writing – review & editing, Supervision, Project administration, Funding acquisition, Conceptualization.

## Declaration of competing interest

The authors declare that they have no known competing financial interests or personal relationships that could have appeared to influence the work reported in this paper.
